# Supplementary material for: Spatial transcriptomics reveals distinct and conserved tumor core and edge architectures that predict survival and targeted therapy response
Source: Nat Commun. 2023 Aug 18;14:5029. doi: 10.1038/s41467-023-40271-4 (PMC10439131; doi:10.1038/s41467-023-40271-4)
Supplement: Supplementary file 1 — Supplementary Information [file 41467_2023_40271_MOESM1_ESM.pdf]

Supplementary Table 1
Patient clinical and demographic information for all 12 samples used for analysis. Abbreviations: mod, moderate; diff, differentiation; Sx, surgery; RT, radiation therapy; TIL, tumor-infiltrating lymphocytes.

Sample ID	Patient ID	Smoker Status (Years Smoked)	Alcohol Consumption	Therapy	Stage	Phenotype	TNM Staging	Differentiation	Extracapsular Spread	Lymphovascular Invasion?	Perineural Invasion?	Bone Invasion?	Tumor Site	Age Range
S1	P1	Current Smoker (15)	Mod Drinker	Sx alone	IVA	TIL	T2N2bM0	mod diff	No	Yes	Yes	N/A	ANTERIOR TONGUE	50-59
S2	P2	Current Smoker (NA)	Previous Drinker	Sx + RT	IVA	Peritumoral	T4aN1M0	mod diff	No	Yes	Yes	No	POSTERIOR FLOOR OF MOUTH	50-59
S3	P3	Ex Smoker (17)	Heavy Drinker	Sx alone	I	Peritumoral	T1N0M0	well diff	N/A	No	No	No	CENTRE LEFT TONGUE	50-59
S4	P4	Never Smoked (0)	Never Drinker	Sx + CRT	IVA	Peritumoral	T2N2bM0	well diff	No	No	No	No	CENTRAL PARTS OF LEFT TONGUE	30-39
S5	P5	Never Smoked (0)	Light Drinker	Sx alone	II	Peritumoral	T2N0M0	mod diff	N/A	Yes	No	No	RIGHT TONGUE	60-69
S6	P6	Never Smoked (0)	Never Drinker	Sx alone	II	Peritumoral	T2N0M0	mod diff	N/A	No	Yes	N/A	CENTRAL PARTS OF LEFT TONGUE	40-49
7	P7	Never Smoked (0)	Mod Drinker	Sx + RT	IVA	TILPeri	T4aN0M0	poor diff	N/A	Yes	Yes	Yes	POSTERIOR LEFT MANDIBLE	70-79
S8	P7	Never Smoked (0)	Mod Drinker	Sx + RT	IVA	TILPeri	T4aN0M0	poor diff	N/A	Yes	Yes	Yes	LATERAL LEFT MANDIBLE	70-79
S9	P8	Current Smoker (NA)	Previous Drinker	Sx + RT	IVA	TILPeri	T4aN1M0	mod diff	No	Yes	Yes	No	ANTERIOR FLOOR OF MOUTH	50-59
S10	P8	Current Smoker (NA)	Previous Drinker	Sx + RT	IVA	TILPeri	T4aN1M0	mod diff	No	Yes	Yes	No	ANTERIOR FLOOR OF MOUTH	50-59
S11	P9	Current Smoker (50)	Heavy Drinker	Sx alone	II	Absent	T2N0M0	mod diff	N/A	No	No	N/A	FLOOR OF MOUTH	60-69
S12	P10	Current Smoker (40)	Heavy Drinker	Sx + CRT	IVA	Absent	T4aN2bM0	mod diff	No	No	No	Yes	FLOOR OF MOUTH	60-69

Supplementary Table 2:
Machine learning classifier 10-fold cross validation statistics, detailing ROC, sensitivity, and specificity values for TC, LE, transitory, and noncancer spots. Abbreviations: svmRadial, support vector machine with radial basis function; avNNet, neural network using model averaging; ROC, receiver operating characteristic; sens, sensitivity; spec, specificity.

Cell type	number cells	Features	Method	ROC	Sens	Spec
core	1929	20	svmRadial	0.991	0.82	0.989
edge	7002	20	svmRadial	0.922	0.694	0.921
noncancer	10926	20	svmRadial	0.943	0.84	0.903
transitory	5019	20	avNNet	0.958	0.755	0.952

Supplementary Figure 1: Cellular deconvolution and annotation of TC and LE across all 12 tissue samples.

a-l. Pathologist annotations, and final TC and LE annotations for samples 1-12; gene-set scores for the TC and LE are visualized for spots with a score greater than the first quartile and median respectively. **m.** Barplot visualizing the cumulative proportion of pathologist annotations. **n.** Consensus tumor copy number segments inferred by numbat through by averaging copy number intensities. **o.** Barplot visualizing malignant and non-malignant spots across samples. **p.** Barplot visualizing the cumulative cell-type deconvolution proportions in each sample. Abbreviations: SCC, squamous cell carcinoma; Tregs, T-regulatory cell.

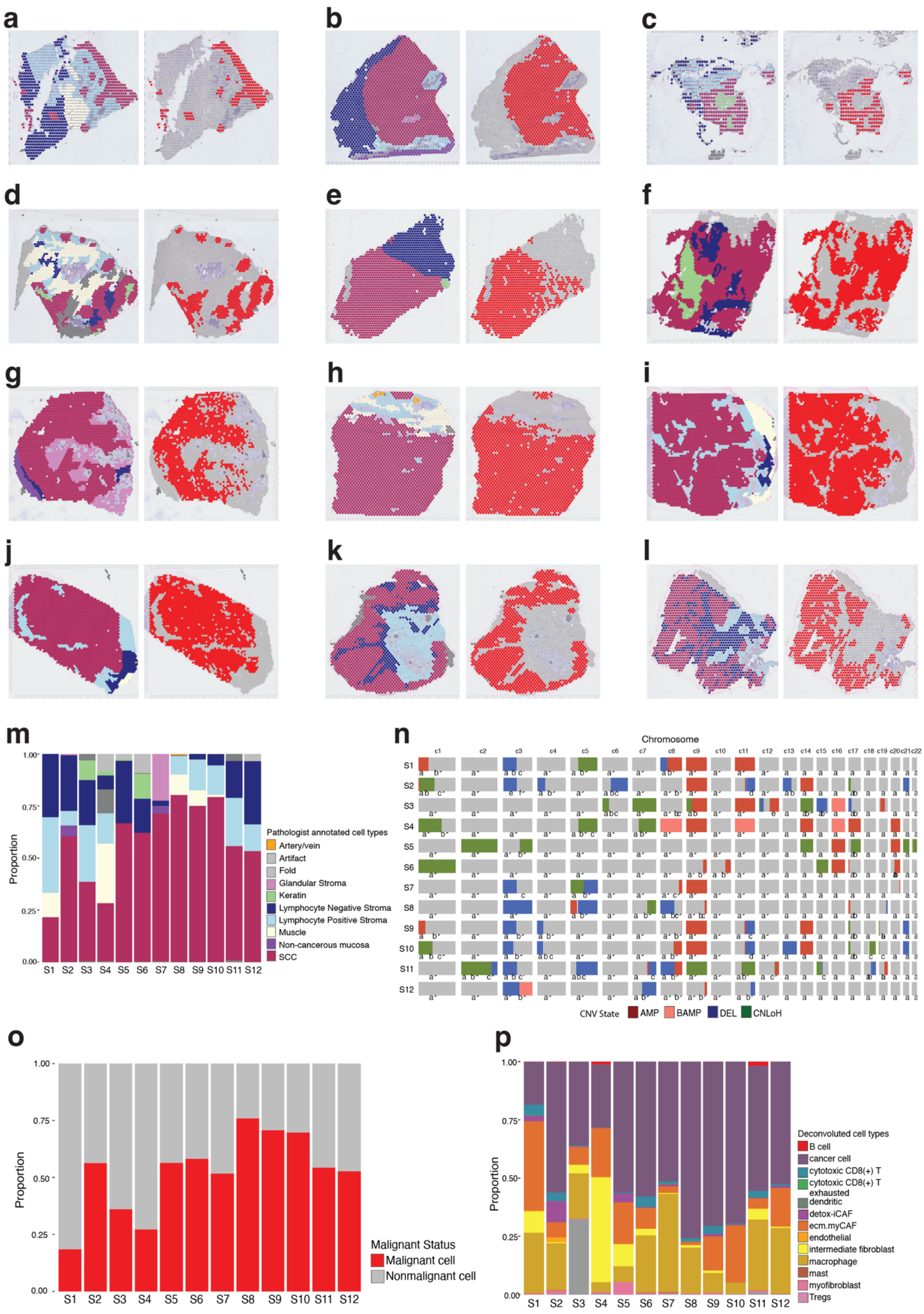

a
b
c
d
e
f
g
h
i
j
k
l
m
Proportion
S1 S2 S3 S4 S5 S6 S7 S8 S9 S10 S11 S12
Pathologist annotated cell types
Artery/vein
Artifact
Fold
Glandular Stroma
Keratin
Lymphocyte Negative Stroma
Lymphocyte Positive Stroma
Muscle
Non-cancerous mucosa
SCC
n
Chromosome
c1 c2 c3 c4 c5 c6 c7 c8 c9 c10 c11 c12 c13 c14 c15 c16 c17 c18 c19 c20 c21 c22
S1 S2 S3 S4 S5 S6 S7 S8 S9 S10 S11 S12
CNV State
AMP
BAMP
DEL
CNLoH
o
Proportion
S1 S2 S3 S4 S5 S6 S7 S8 S9 S10 S11 S12
Malignant Status
Malignant cell
Nonmalignant cell
p
Proportion
S1 S2 S3 S4 S5 S6 S7 S8 S9 S10 S11 S12
Deconvoluted cell types
B cell
cancer cell
cytotoxic CD8(+) T
cytotoxic CD8(+) T exhausted
dendritic
detox-iCAF
ecm.myCAF
endothelial
intermediate fibroblast
macrophage
mast
myofibroblast
Tregs

Supplementary Figure 2: TC and LE regions are enriched in unique biological processes and upstream regulators

a-l. Final TC and LE annotations for samples 1-12. **m.** Barplot visualizing the proportion of annotated TC, transitory, and LE spots across samples. **n.** Comparative expression of six ge ne-sets across the TC, LE, and other squamous cell carcinoma (SCC) spots. (For all gene set comparison plots, significance of differences between the TC and LE was determined using a paired T test. Circles are representative of mean and lines are standard deviation. All tested gene-sets had a significant p-value, *p < 0.05, **p < 0.01, ***p < 0.001, ****p < 0.0001.). **o.** Hallmark pathway screening plot of 8,899 pathways and their enrichment in the TC and LE. Pathways were tested for significance using multivariate distribution testing with a Bonferroni correction applied **p.** Barplot displaying the cumulative average logFC for differentially expressed transcription factors between the TC and LE across more than 9 samples, inferred by SCENIC. **q.** IPA heatmap visualizing predicted activation and deactivation of upstream regulatory molecules. Upstream regulators are displayed if they are activated or deactivated across 10 samples and ordered based on similarity of z-score for each pathway across samples. **n.** Comparative expression of four gene-sets across the TC, LE, and other squamous cell carcinoma (SCC) spots. For all gene set comparison plots, significance of differences between the TC and LE was determined using a two-sided paired T test and corrected with a Bonferroni correction. Circles are representative of mean and lines are standard deviation. All tested gene-sets had a significant adjusted p-value, *p < 0.05, **p < 0.01, ***p < 0.001, ****p < 0.0001, Bonferroni correction). P-values for cell cycle = 2e-04, angiogenesis = 6.2e-04, EMT = 0.041, epithelial differentiation = 4.4e-06, P-EMT = 2e-04 (n=12 samples across 10 independent patients). Abbreviations: TC, tumor core; LE, leading edge; KEGG, Kyoto Encyclopedia of Genes and genomes; EMT, epithelial-mesenchymal transition; epithelial diff, epithelial differentiation; P-EMT, partial epithelial-mesenchymal transition.

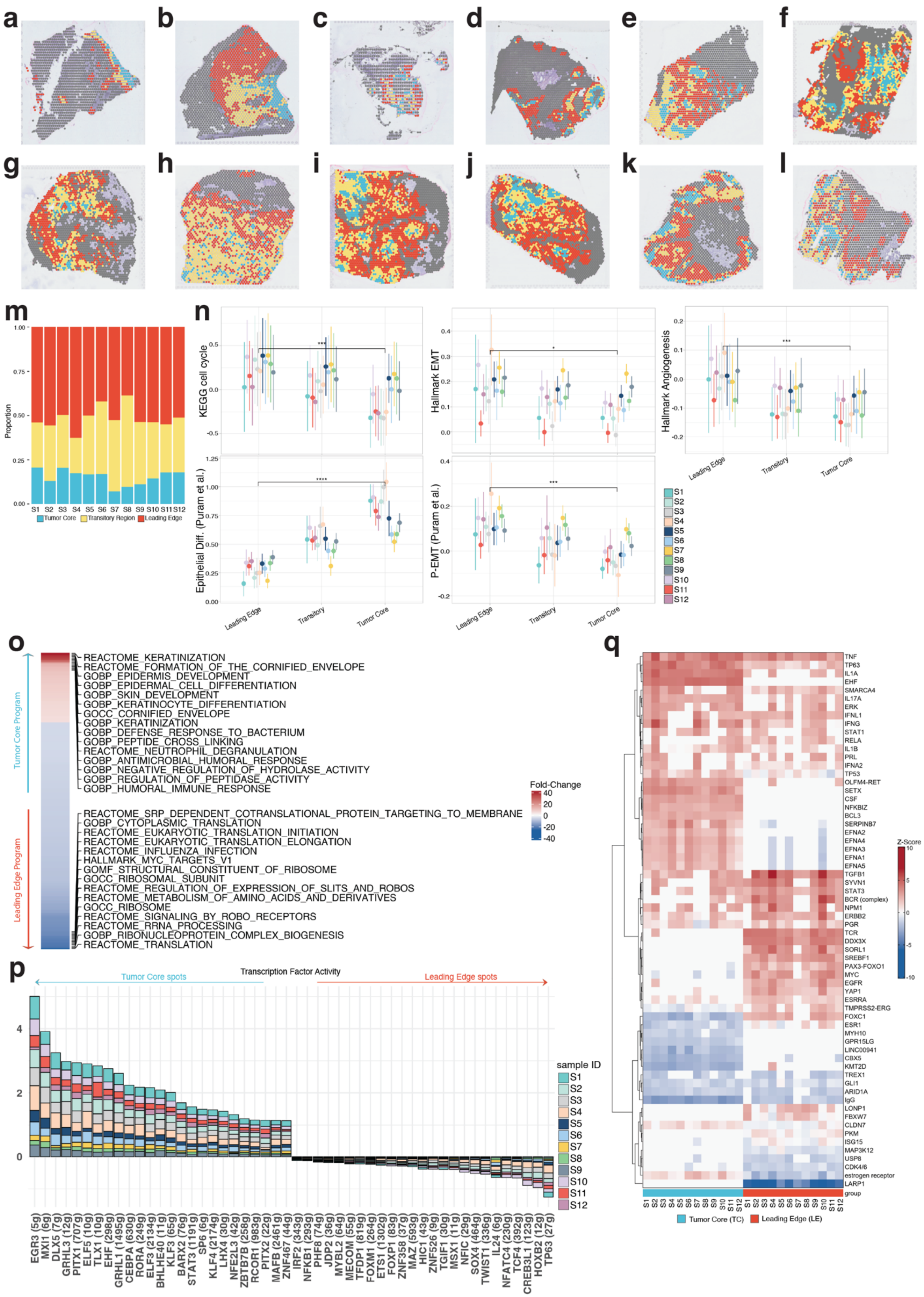
a
b
c
d
e
f
g
h
i
j
k
l
m
Proportion
S1 S2 S3 S4 S5 S6 S7 S8 S9 S10 S11 S12
Tumor Core
Transitory Region
Leading Edge
n
KEGG cell cycle
Hallmark EMT
Hallmark Angiogenesis
Epithelial Diff. (Puram et al.)
P-EMT (Puram et al.)
Leading Edge
Transitory
Tumor Core
o
Tumor Core Program
Leading Edge Program
REACTOME_KERATINIZATION
REACTOME_FORMATION_OF_THE_CORNIFIED_ENVELOPE
GOBP_EPIDERMIS_DEVELOPMENT
GOBP_EPIDERMAL_CELL_DIFFERENTIATION
GOBP_SKIN_DEVELOPMENT
GOBP_KERATINOCYTE_DIFFERENTIATION
GOCC_CORNIFIED_ENVELOPE
GOBP_KERATINIZATION
GOBP_DEFENSE_RESPONSE_TO_BACTERIUM
GOBP_PEPTIDE_CROSS_LINKING
REACTOME_NEUTROPHIL_DEGRANULATION
GOBP_ANTIMICROBIAL_HUMORAL_RESPONSE
GOBP_NEGATIVE_REGULATION_OF_HYDROLASE_ACTIVITY
GOBP_REGULATION_OF_PEPTIDASE_ACTIVITY
GOBP_HUMORAL_IMMUNE_RESPONSE
REACTOME_SRP_DEPENDENT_COTRANSLATIONAL_PROTEIN_TARGETING_TO_MEMBRANE
GOBP_CYTOPLASMIC_TRANSLATION
REACTOME_EUKARYOTIC_TRANSLATION_INITIATION
REACTOME_EUKARYOTIC_TRANSLATION_ELONGATION
REACTOME_INFLUENZA_INFECTION
HALLMARK_MYC_TARGETS_V1
GOMF_STRUCTURAL_CONSTITUENT_OF_RIBOSOME
GOCC_RIBOSOMAL_SUBUNIT
REACTOME_REGULATION_OF_EXPRESSION_OF_SLITS_AND_ROBOS
REACTOME_METABOLISM_OF_AMINO_ACIDS_AND_DERIVATIVES
GOCC_RIBOSOME
REACTOME_SIGNALING_BY_ROBO_RECEPTORS
REACTOME_RRNA_PROCESSING
GOBP_RIBONUCLEOPROTEIN_COMPLEX_BIOGENESIS
REACTOME_TRANSLATION
Fold-Change
p
Transcription Factor Activity
Tumor Core spots
Leading Edge spots
sample ID
EGR3 (5g)
MXI1 (6g)
DLX5 (7g)
GRHL3 (12g)
PITX1 (707g)
ELF5 (10g)
TLX1 (10g)
EHF (298g)
GRHL1 (1495g)
CEBPA (630g)
RORA (2134g)
ELF3 (249g)
BHLHE40 (11g)
KLF3 (65g)
BARX2 (76g)
STAT3 (1191g)
SP6 (6g)
KLF4 (2174g)
LHX4 (30g)
NFE2L3 (42g)
ZBTB7B (258g)
RCOR1 (983g)
PITX2 (22g)
MAFB (2461g)
ZNF467 (44g)
IRF2 (343g)
NFKB1 (293g)
PHF8 (74g)
JDP2 (36g)
MYBL2 (64g)
MECOM (55g)
TFDP1 (819g)
FOXM1 (264g)
ETS1 (1302g)
FOXP1 (83g)
ZNF358 (37g)
MAZ (593g)
HIC1 (43g)
ZNF526 (9g)
TGIF1 (30g)
MSX1 (11g)
NFIC (29g)
SOX4 (464g)
TWIST1 (336g)
IL24 (6g)
NFATC4 (230g)
TCF4 (392g)
CREB3L1 (123g)
HOXB2 (12g)
TP63 (27g)
q
TNF
TP63
IL1A
EHF
SMARCA4
IL17A
ERK
IFNL1
IFNG
STAT1
RELA
IL1B
PRL
IFNA2
TP53
OLFM4-RET
SETX
CSF
NFKBIZ
BCL3
SERPINB7
EFNA2
EFNA4
EFNA3
EFNA1
EFNA5
TGFB1
SYVN1
STAT3
BCR (complex)
NPM1
ERBB2
PGR
TCR
DDX3X
SORL1
SREBF1
PAX3-FOXO1
MYC
EGFR
YAP1
ESRRA
TMPRSS2-ERG
FOXC1
ESR1
MYH10
GPR15LG
LINC00941
CBX5
KMT2D
TREX1
GLI1
ARID1A
IgG
LONP1
FBXW7
CLDN7
PKM
ISG15
MAP3K12
USP8
CDK4/6
estrogen receptor
LARP1
group
Z-Score
Tumor Core (TC)
Leading Edge (LE)

Supplementary Figure 3: Transcriptional heterogeneity in TC and LE regions is not explained by HNSCC molecular subtypes and is characterized by unique cell-cell communication interactions

a. Stacked bar plot visualizing HNSCC molecular subtype deconvolution scores for TC, LE, and other SCC spots across samples. Deconvolution scores were obtained through integration and label transfer with 275 HPV negative OSCC bulk RNA sequencing samples labeled by subtype from the NCI TCGA. **b-c.** Bar plot displaying single sample gene-set enrichment scores for the TC (p-value = 3.9e-10) and LE (p-value = 0.005) gene-sets across TCGA HNSCC molecular subtype data (n=275 biologically independent HPV negative OSCC samples). Significance was compared between groups using a Kruskal-Wallis one-way analysis of variance test. *$p < 0.05$, **$p < 0.01$, ***$p < 0.001$, ****$p < 0.0001$. Box spans 25th-75th percentiles, center line indicates median, whiskers extend to minima and maxima within 1.5*IQR, and outliers are individually plotted. **d.** Stacked bar plot visualizing proportion of tumor-specific subclones inferred by CNV probability across different OSCC samples. **e.** Micrograph image of CD24 and CD44 immunofluorescence staining from three independent OSCC patient samples. Scale bar for 100 µm provided in the top left image. **f.** Outgoing and incoming signaling pathway strength for TC cancer cells and LE cancer cells. Signaling pathways are ordered based on strength of interaction. **g.** Bar plot visualizing number and cumulative strength of inferred ligand-receptor interactions for spatially deconvolved LE ecm-myCAFs with LE cancer cells, TC cancer cells with TC cancer cells, and LE cancer cells with LE cancer cells. Interaction strength represents the probability of ligand-receptor interaction. **h.** Circos plots describing relative strength and direction of extracellular interaction among TC and macrophage cells during desmosome and CDH pathway signaling. **i.** Circos plots describing the relative strength and direction of extracellular interaction among LE, macrophage, ecm.myCAF, and cytotoxic (CD8+) T cells during laminin and collagen pathway signaling. Abbreviations: HNSCC, head and neck squamous cell carcinoma; TCGA, The Cancer Genome Atlas; TC, tumor core; LE, leading edge.

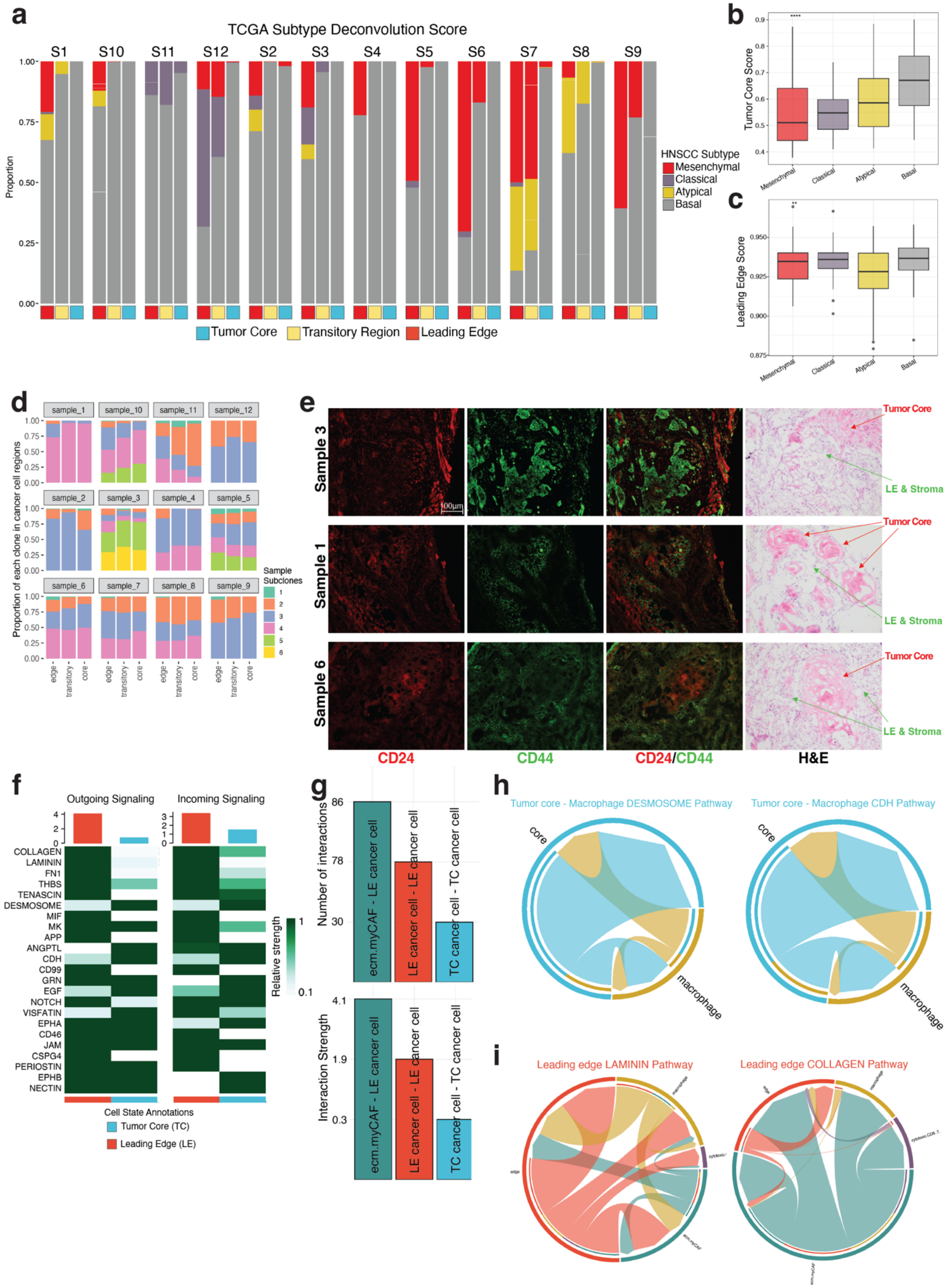
a
TCGA Subtype Deconvolution Score
S1 S10 S11 S12 S2 S3 S4 S5 S6 S7 S8 S9
Proportion
HNSCC Subtype
Mesenchymal
Classical
Atypical
Basal
Tumor Core
Transitory Region
Leading Edge
b
Tumor Core Score
Mesenchymal Classical Atypical Basal
c
Leading Edge Score
d
Proportion of each clone in cancer cell regions
sample_1 sample_10 sample_11 sample_12
sample_2 sample_3 sample_4 sample_5
sample_6 sample_7 sample_8 sample_9
Sample Subclones
edge transitory core
e
Sample 3
Sample 1
Sample 6
Tumor Core
LE & Stroma
100μm
CD24
CD44
CD24/CD44
H&E
f
Outgoing Signaling
Incoming Signaling
COLLAGEN
LAMININ
FN1
THBS
TENASCIN
DESMOSOME
MIF
MK
APP
ANGPTL
CDH
CD99
GRN
EGF
NOTCH
VISFATIN
EPHA
CD46
JAM
CSPG4
PERIOSTIN
EPHB
NECTIN
Relative strength
Cell State Annotations
Tumor Core (TC)
Leading Edge (LE)
g
Number of interactions
86 78 30
ecm.myCAF - LE cancer cell
LE cancer cell - LE cancer cell
TC cancer cell - TC cancer cell
Interaction Strength
4.1 1.9 0.3
h
Tumor core - Macrophage DESMOSOME Pathway
Tumor core - Macrophage CDH Pathway
core
macrophage
i
Leading edge LAMININ Pathway
Leading edge COLLAGEN Pathway

Supplementary Figure 4: ScPred annotation of TC and LE states across 29 spatial samples.

Stained tissue section (left), scPred classification on stained tissue (middle), and a UMAP colored by scPred classification (right) for spatial transcriptomics samples from **a.** CESC and CHC **b.** COAD and cSCC **c.** cSCC and GBM **d.** IDC, ILC and HCC **e.** HCC and ICC **f.** COAD and Lung SCC, SKCM **g.** EC and cSCC **h.** PDAC **i.** PRAD and MB **j.** MB and CNS embryonal. Abbreviations: cSCC, cutaneous squamous cell carcinoma; SCC, squamous cell carcinoma; ICC, intrahepatic cholangiocarcinoma; PRAD, prostate adenocarcinoma; PDAC, pancreatic ductal carcinoma; CESC, cervical squamous cell carcinoma; COAD, colon adenocarcinoma; IDC, invasive carcinoma; HCC, hepatocellular carcinoma, SKCM, skin cutaneous melanoma; ILC, invasive lobular carcinoma; EC, endometrial cancer; CHC, combined hepatocellular and cholangiocarcinoma; GBM, glioblastoma multiforme; CNS embryonal, central nervous system embryonal tumor; MB, medulloblastoma.

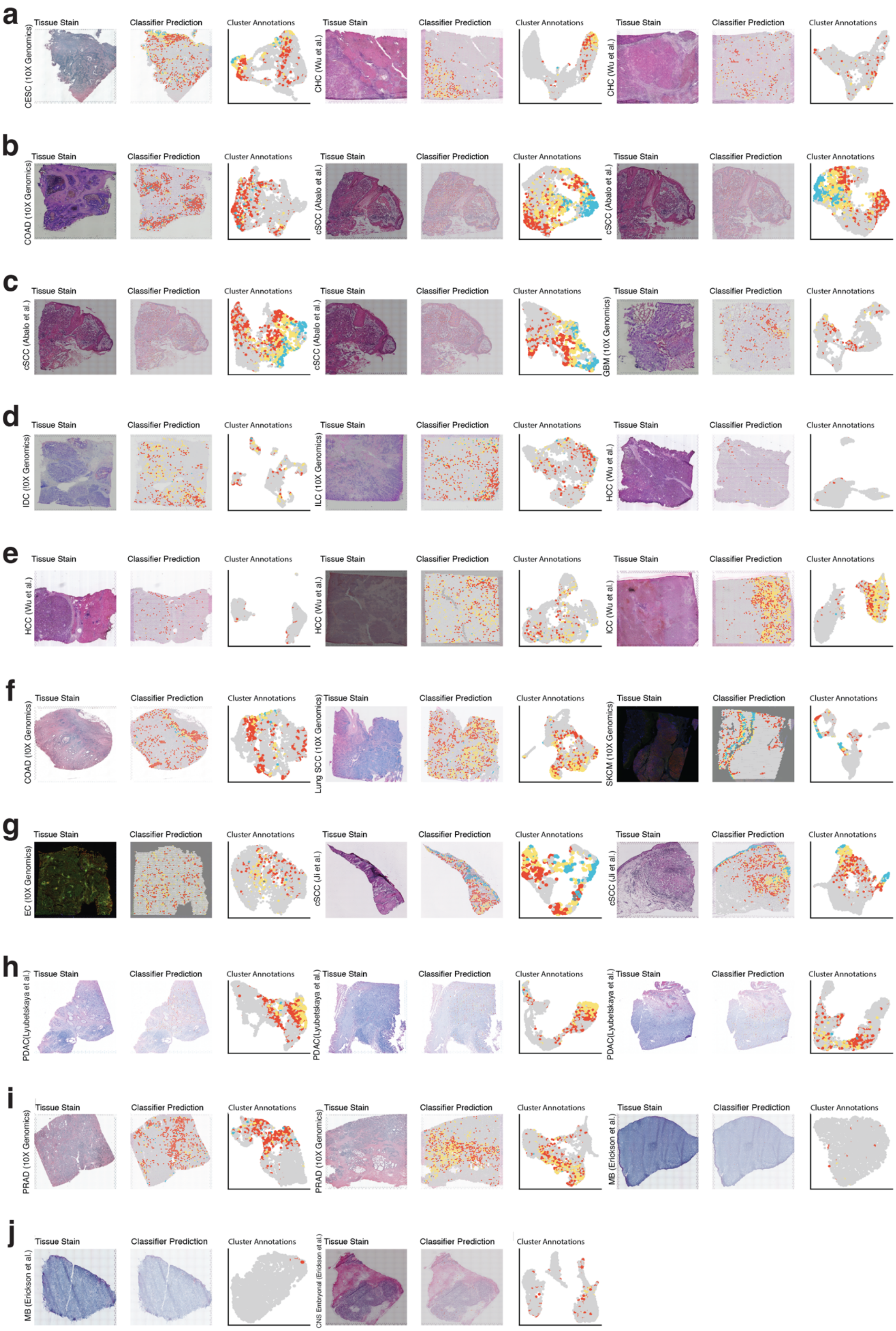

a
Tissue Stain
Classifier Prediction
Cluster Annotations
CESC (10X Genomics)
CHC (Wu et al.)
CHC (Wu et al.)
b
COAD (10X Genomics)
cSCC (Abalo et al.)
cSCC (Abalo et al.)
c
cSCC (Abalo et al.)
cSCC (Abalo et al.)
GBM (10X Genomics)
d
IDC (10X Genomics)
ILC (10X Genomics)
HCC (Wu et al.)
e
HCC (Wu et al.)
HCC (Wu et al.)
ICC (Wu et al.)
f
COAD (10X Genomics)
Lung SCC (10X Genomics)
SKCM (10X Genomics)
g
EC (10X Genomics)
cSCC (Ji et al.)
cSCC (Ji et al.)
h
PDAC(Lyubetskaya et al.)
PDAC(Lyubetskaya et al.)
PDAC(Lyubetskaya et al.)
i
PRAD (10X Genomics)
PRAD (10X Genomics)
MB (Erickson et al.)
j
MB (Erickson et al.)
CNS Embryonal (Erickson et al.)

Supplementary Figure 5: Core and edge regions are correlated to patient outcomes
a-b. Boxplots comparing TC and LE gene-set single sample gene-set enrichment scores across pathological tumor stages among 247 OSCC samples. Significance was compared between groups using a Kruskal-Wallis one-way analysis of variance test. Box spans 25th-75th percentiles, center line indicates median, whiskers extend to minima and maxima within 1.5*IQR, and outliers are individually plotted. **c.** Overall survival and disease specific survival OSCC outcomes for TC and LE gene-set enrichment scores in a validation dataset. P-values and hazard ratios displayed were calculated using a cox proportional-hazard regression. **d.** Correlation plot visualizing TC geneset score relative to LE geneset score among 275 OSCC samples, derived from a Pearson correlation with a two-sided t-distribution. Grey band represents a 95% confidence interval. **e.** Progression free interval pan-cancer outcome for TC and LE gene-set enrichment scores. P-values and hazard ratios displayed were calculated using a cox proportional-hazard regression. **f.** Correlation plot visualizing EPIC CAF geneset score relative to LE geneset score among 275 OSCC samples, derived from a Pearson correlation with a two-sided t-distribution. Grey band represents a 95% confidence interval. Abbreviations: OS, overall survival; DSS, disease-specific survival; THCA, thyroid carcinoma; STAD, stomach adenocarcinoma; SKCM, skin cutaneous melanoma; SARC, sarcoma; PRAD, prostate adenocarcinoma; PAAD, pancreatic adenocarcinoma; OV, ovarian serous cystadenocarcinoma; OSCC, oral squamous cell carcinoma; MESO, mesothelioma; LUSC, lung squamous cell carcinoma; LUAD, lung adenocarcinoma; LIHC, liver hepatocellular carcinoma; KIRP, kidney renal papillary cell carcinoma; KIRC, kidney renal clear cell carcinoma; GBMLGG, brain lower grade glioma and glioblastoma multiforme; COADREAD, colon adenocarcinoma/rectal adenocarcinoma; CESC, cervical squamous cell carcinoma; BRCA, breast invasive carcinoma; BLCA, bladder urothelial carcinoma; ACC, adrenocortical carcinoma; CAF, cancer-associated fibroblast.

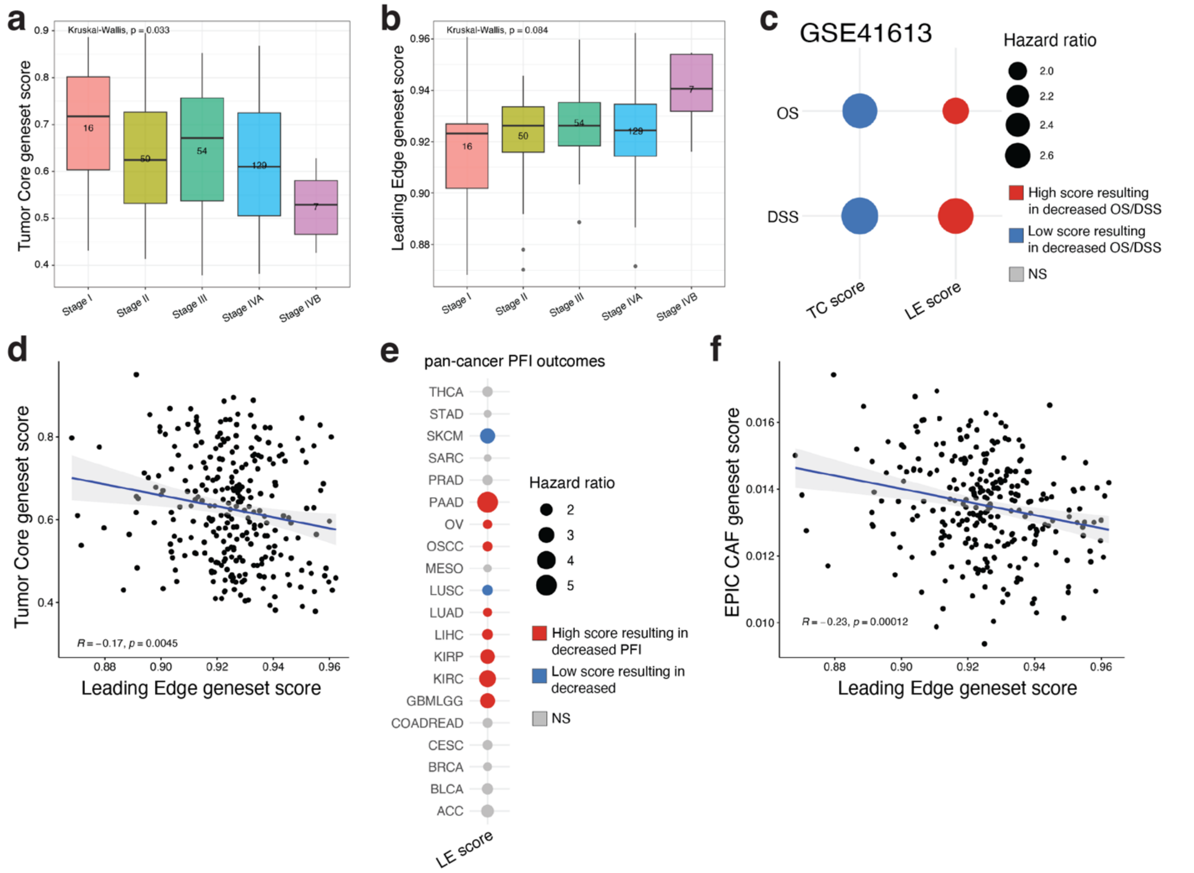

Supplementary Figure 6: Splicing dynamics and drug responses of spatially unique cancer cells

a. Phase portraits showing the ratio of spliced and unspliced RNA ratios for top differentially spliced genes, purple lines depict splicing steady state. **b.** UMAP and state diagram showing the resultant vector field following an in-silico perturbation of CD274 (PD-L1). **c.** UMAP and state diagram showing the resultant vector field following an in-silico perturbation of CTLA4. **d.** Boxplot comparing core incoming vector field strengths between high AAC and low AAC drugs stratified based on median. AAC groupings are compared using a two-sided Wilcoxon rank sum test (n=70 independent drugs). Box spans 25th-75th percentiles, center line indicates median, whiskers extend to minima and maxima within 1.5*IQR. **e,f** Core incoming and edge outgoing vector field strengths compared across drug classes using a Kruskal-Wallis one way analysis of variance test (n=46 independent drugs). Box spans 25th-75th percentiles, center line indicates median, whiskers extend to minima and maxima within 1.5*IQR.

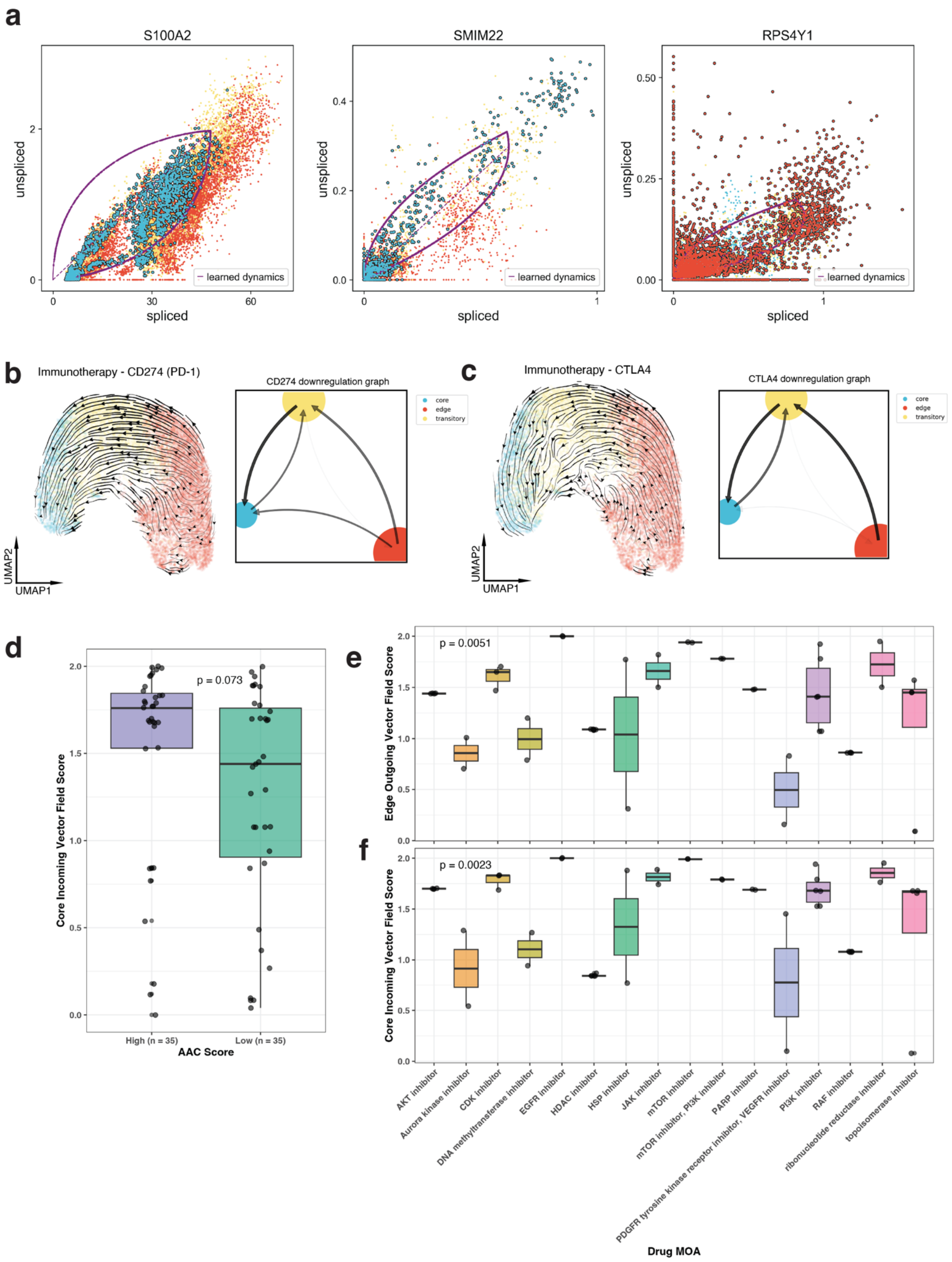

a
S100A2
SMIM22
RPS4Y1
unspliced
spliced
learned dynamics
b
Immunotherapy - CD274 (PD-1)
CD274 downregulation graph
core
edge
transitory
UMAP2
UMAP1
c
Immunotherapy - CTLA4
CTLA4 downregulation graph
d
p = 0.073
Core Incoming Vector Field Score
High (n = 35)
Low (n = 35)
AAC Score
e
p = 0.0051
Edge Outgoing Vector Field Score
f
p = 0.0023
Core Incoming Vector Field Score
AKT inhibitor
Aurora kinase inhibitor
CDK inhibitor
DNA methyltransferase inhibitor
EGFR inhibitor
HDAC inhibitor
HSP inhibitor
JAK inhibitor
mTOR inhibitor
mTOR inhibitor, PI3K inhibitor
PARP inhibitor
PDGFR tyrosine kinase receptor inhibitor, VEGFR inhibitor
PI3K inhibitor
RAF inhibitor
ribonucleotide reductase inhibitor
topoisomerase inhibitor
Drug MOA
