# Supplementary material for: Spatial transcriptomics reveals distinct and conserved tumor core and edge architectures that predict survival and targeted therapy response
Source: Nat Commun. 2023 Aug 18;14:5029. doi: 10.1038/s41467-023-40271-4 (PMC10439131; doi:10.1038/s41467-023-40271-4)
Supplement: Supplementary file 3 — Description of Additional Supplementary Files [file 41467_2023_40271_MOESM3_ESM.pdf]

Description of Additional Supplementary Files

File Name: Supplementary Data 1
Description: Differentially expressed genes identified between all three nodal clusters of cancer cells. Genes were identified using a two-sided Wilcox rank sum test with a Bonferroni correction.

File Name: Supplementary Data 2
Description: Comprehensive hallmark pathway screening results for TC and LE spots. Table contains results from an two sided multivariate distribution test, p-values are adjusted using a Bonferroni correction.

File Name: Supplementary Data 3
Description: Differentially expressed transcription factors between TC and LE spots. Table contains log2FC and adjusted p-value information for all differentially expressed genes as determined through a two-sided Wilcox rank sum test with a Bonferroni correction.

File Name: Supplementary Data 4
Description: DEGs factors between spatially deconvolved TC and LE cancer cells. Table contains log2FC and adjusted p-value information for all differentially expressed genes as determined through a two-sided Wilcox rank sum test with a Bonferroni correction.

File Name: Supplementary Data 5
Description: Cell chat-predicted cell-cells signaling ligand receptor pairs and signaling strength (interaction probability). Table contains LE cancer cells, TC cancer cells, and LE ecm-myCAF cells as both sources and targets of signaling.

File Name: Supplementary Data 6
Description: Differentially spliced genes identified with scVelo are ordered based on fit likelihood. Genes differentially spliced based on LE, TC, and transitory spots are indicated.

File Name: Supplementary Data 7
Description: Trimmed AAC means for 70 drugs, drug mechanism of action, drug indication, upregulated and downregulated gene targets of drugs, and resultant dynamo in silico based perturbation vector field quantification.
