# Supplementary material for: Spatial transcriptomics reveals distinct and conserved tumor core and edge architectures that predict survival and targeted therapy response
Source: Nat Commun. 2023 Aug 18;14:5029. doi: 10.1038/s41467-023-40271-4 (PMC10439131; doi:10.1038/s41467-023-40271-4)
Supplement: Supplementary file 11 — Reporting Summary [file 41467_2023_40271_MOESM11_ESM.pdf]

nature portfolio

Corresponding author(s): Pinaki Bose

Last updated by author(s): June 28, 2023

Reporting Summary

Nature Portfolio wishes to improve the reproducibility of the work that we publish. This form provides structure for consistency and transparency in reporting. For further information on Nature Portfolio policies, see our Editorial Policies and the Editorial Policy Checklist.

Statistics

For all statistical analyses, confirm that the following items are present in the figure legend, table legend, main text, or Methods section.

n/a	Confirmed	
☐	☒	The exact sample size (n) for each experimental group/condition, given as a discrete number and unit of measurement
☐	☒	A statement on whether measurements were taken from distinct samples or whether the same sample was measured repeatedly
☐	☒	The statistical test(s) used AND whether they are one- or two-sided<br>*Only common tests should be described solely by name; describe more complex techniques in the Methods section.*
☐	☒	A description of all covariates tested
☐	☒	A description of any assumptions or corrections, such as tests of normality and adjustment for multiple comparisons
☐	☒	A full description of the statistical parameters including central tendency (e.g. means) or other basic estimates (e.g. regression coefficient) AND variation (e.g. standard deviation) or associated estimates of uncertainty (e.g. confidence intervals)
☐	☒	For null hypothesis testing, the test statistic (e.g. F, t, r) with confidence intervals, effect sizes, degrees of freedom and P value noted<br>*Give P values as exact values whenever suitable.*
☒	☐	For Bayesian analysis, information on the choice of priors and Markov chain Monte Carlo settings
☒	☐	For hierarchical and complex designs, identification of the appropriate level for tests and full reporting of outcomes
☐	☒	Estimates of effect sizes (e.g. Cohen's d, Pearson's r), indicating how they were calculated

Our web collection on statistics for biologists contains articles on many of the points above.

Software and code

Policy information about availability of computer code

Data collection	Spatial transcriptomics was performed on OSCC cryosections with the Visium Spatial Gene Expression Slide & Reagent Kit, 16 rxns PN-1000184. according to the manufacturer's protocol (10x Genomics, Pleasanton, CA, USA). Briefly, OCT-embedded 10 micrometer-thick cryosections of OSCC samples were placed on the Visium spatial slide. Sections were enzymatically permeabilized for 24 min. cDNA was obtained from mRNA bound to capture oligos printed on the slide. cDNA quantification was performed using Agilent Bioanalyzer High Sensitivity Kit (5067-4626) on an Agilent Bioanalyzer 2100 (Agilent Technologies, CA, USA). cDNA libraries were sequenced on an Illumina NovaSeq 6000 sequencer using the SP flowcell (200 cycles) at the Centre for Health Genomics and Informatics (CHGI, University of Calgary, Alberta, Canada).
Data analysis	Sequencing reads were aligned using the 10x Genomics Space Ranger 1.3.1 pipeline to the standard GRCh38 reference genome.<br><br>Spatial transcriptomics data was processed in R using the package Seurat (v4.2.0) for normalization, quality control, batch effect correction, dimensionality reduction, Louvain clustering and state annotation, differential expression analysis, and gene set scoring. The 'CARD' R package (Version 1.0) was used to perform deconvolution of spatial transcriptomic spots into single-cell cell types with the 'CARD_deconvolution' function. The 'numbat' R package (Version 1.1.0) was used to conduct haplotype-aware CNV inference on all 12 spatial transcriptomics objects from raw ST BAM files. The ape R package (Version 5.6-2) was used to visualize hierarchical states of cancer cell identity. Differentially expressed genes between nodal clusters were visualized with the 'SCpubr' R package (Version 1.1.2.9000). Stacked bar plots showing cumulative expression log2FC for each gene across all samples were generated using an adaptation of the constructConsensus function and available at our Zenodo github DOI Zenodo DOI 10.5281/zenodo.8079095. Differentially expressed genes for each sample were imported into Ingenuity Pathway Analysis for pathway enrichment analysis. Ingenuity pathway analysis exports were imported into the multienrichjam R package (Version 0.0.57.9). Correlation heatmaps comparing similarity of transcriptional profiles were generated using the R package ComplexHeatmap (v2.14.0). The 'ggplot2' (Version 3.4.0) and 'ggrepl' R packages (Version 0.9.2) were used to vizualize differential

pathway activity. The R package 'Nebulosa' was used in combination with the 'SCpubr' R package (Version 1.1.2.9000) to visualize kernel density estimates for module scores and individual genes. Regulatory network inference and clustering was run using the pySCENIC python package (v0.12.1) to infer transcription factor activity.

TCGA transcriptomic and survival data was downloaded and analyzed using the UCSCXenaTools R package (v1.4.8). Gene set enrichment scores were calculated using the 'singscore' R package (v1.16.0) and plotted using the survminer R package (v0.4.9). Cox proportional hazard testing for survival analysis in the TCGA data was performed using the R 'survival' package (v3.3-1). Benjamini-Hochberg corrections were applied with the 'stats' R package (Version 4.2.2). Pearson correlation test scores in our TCGA data was conducted using the 'ggpubr' (Version 0.5.0) 'stat_cor' function and plotted using the 'ggscatter' function. A CAF score was identified by using 'EPIC' based deconvolution in the 'immunedeconv' R package (Version 1.1.5) with default settings. Validation intensity data was preprocessed and background corrected using the 'limma' R package (Version 3.54.1). TCGA subtype data for 279 OSCC samples was downloaded using the 'PanCancerAtlas_subtypes' function from the 'TCGAbiolinks' R package (Version 2.25.3).Machine learning models for characterization of tumoral states were generated using the scPred R package (v.1.92).

The CellChat package (v.1.4.0) was used to infer cell-cell interaction networks from Seurat objects. Cell neighbors were identified using code available at Zenodo github DOI Zenodo DOI 10.5281/zenodo.8079095.

The command line interface for the python package velocyto (v0.6) was used to identify spliced and unspliced reads. The python package scvelo (v0.2.5) was leveraged to infer developmental trajectories based on spliced and unspliced reads. IC50 drug response in HPV negative HNSCC cell lines was identified using the PharmacoGx R package (v3.0.2). drug-gene interactions identified as being upregulated or downregulated from the DGIdb. The python package dynamo (v1.2.0) was utilized to perform In-silico perturbation analysis on gene targets of drugs characterized by high IC50 and low IC50 values. Drug mechanism of action was identified using the PRISM database in the R package 'PharmacoGx' (Version 3.2.0).

The interactive spatial transcriptomic atlas was generated using RShiny (v1.1.0), shinythemes (v.1.1.2), and shinyLP (v.1.1.2) R packages.

For manuscripts utilizing custom algorithms or software that are central to the research but not yet described in published literature, software must be made available to editors and reviewers. We strongly encourage code deposition in a community repository (e.g. GitHub). See the Nature Portfolio guidelines for submitting code & software for further information.

Data

Policy information about availability of data

All manuscripts must include a data availability statement. This statement should provide the following information, where applicable:

- Accession codes, unique identifiers, or web links for publicly available datasets
- A description of any restrictions on data availability
- For clinical datasets or third party data, please ensure that the statement adheres to our policy

The Raw and SpaceRanger processed spatial transcriptomics data generated in this study have been deposited in the National Center of Biotechnology Information's Gene Expression Omnibus (GEO) database under accession code GSE208253 [https://www.ncbi.nlm.nih.gov/geo/query/acc.cgi?acc=GSE208253]. The processed Seurat objects, loom files generated by velocyto, and the scPred prediction model data are available at https://doi.org/10.6084/m9.figshare.20304456.v1. Spatial datasets are also available for public access at our companion portal http://www.pboselab.ca/spatial_OSCC/. In silico perturbation results are available for public access at www.pboselab.ca/dynamo_OSCC. The analyzed spatial transcriptomics differential expression, transcription factor, hallmark pathway, cellChat, differential splicing, and drug perturbation data generated in this study are provided in the Supplementary Information/Source Data file. The Hallmark gene-sets for core-edge testing data used in this study are available in the Molecular Signatures Database v7.5.1.107 The P-emt gene-set and single-cell HNSCC data used for deconvolution data used in this study are available in the GEO database under accession code GSE103332 [https://www.ncbi.nlm.nih.gov/geo/query/acc.cgi?acc=GSE103322]. Cancer stem cell gene-set data used in this study were extracted from literature. TheLifeome, GEO, Mendeley, and 10X Genomics databases under accession codes Lifeome: 7:eabg3750, GEO: bulk RNA-sequencing data and associated clinical data used in this study are available in the National Cancer Institute's The Cancer Genome Atlas database through UCSC Xena [https://xena.ucsc.edu]. The validation genomic survival dataset data used in this study are available in the GEO database under accession code GSE41613 [https://www.ncbi.nlm.nih.gov/geo/query/acc.cgi?acc=GSE41613]. The scpred analysis data used in this study are available in the GSE144240, Mendeley DOI: 10.17632/2bh5fchcv6.1, Mendeley DOI: 10.17632/svw96g68dv.1, GEO: GSE211895 [http://lifeome.net/supp/livercancer-st/data.htm, https://www.ncbi.nlm.nih.gov/geo/query/acc.cgi?acc=GSE144240, https://data.mendeley.com/datasets/2bh5fchcv6/1, https://data.mendeley.com/datasets/svw96g68dv/1, https://www.ncbi.nlm.nih.gov/geo/query/acc.cgi?acc=GSE211895, https://www.10xgenomics.com/resources/datasets]. The drug response data used in this study are available in the PharmacoDB database [https://pharmacodb.ca/]. Source data are provided with this paper.

Human research participants

Policy information about studies involving human research participants and Sex and Gender in Research.

Reporting on sex and gender	Sex and gender were not considered in the study design and determined based on patient medical records. Our study analyzed 10 HPV-negative OSCC (8 Males, 2 Females) patients. This distribution approximately resembles the ratio of males to females for OSCC prevalence in the population. No sex or gender stratified analyses were performed due to a lack of statistical power. Age ranges and clinical characteristics of enrolled individuals is provided in our Supplementary Table 1.
Population characteristics	Our study analyzed data from 10 HPV-negative OSCC (8 Males, 2 Females) patients. Age ranges and clinical characteristics of enrolled individuals is provided in our Supplementary Table 1.
Recruitment	Retrospectively collected samples were used for these analyses. Informed consent for tissue collection and research was obtained from each patient. No participant compensation was provided.

Ethics oversight	This study was approved by the Health Research Ethics Board of Alberta – Cancer Committee (reference number: HREBA.CC-16-0644) and is consistent with the Declaration of Helsinki.

Note that full information on the approval of the study protocol must also be provided in the manuscript.

Field-specific reporting

Please select the one below that is the best fit for your research. If you are not sure, read the appropriate sections before making your selection.

☒ Life sciences ☐ Behavioural & social sciences ☐ Ecological, evolutionary & environmental sciences

For a reference copy of the document with all sections, see nature.com/documents/nr-reporting-summary-flat.pdf

Life sciences study design

All studies must disclose on these points even when the disclosure is negative.

Sample size	Due to the unbiased nature of our investigations, no effect size could be approximated. Consequently, a priori sample size estimation was performed. Sample size was chosen based on availability of samples with sufficient sequencing quality and presence of an OSCC leading edge
Data exclusions	2 collected samples were excluded from analysis due to poor sequencing quality.
Replication	We showed reproducibility of gene expression profiles by generating consensus plots. These plots visualized stacked cumulative log-fold changes of differentially expressed genes, coloured by individual patient contributions. Spatial transcriptomic data was also obtained on serial sections from two patients in our cohort and observed reproducible expression profiles within both patients.<br><br>To validate our survival findings in an external database, data from GSE41613 containing 93 HPV negative OSCC patients was downloaded and imported into R. Data was processed similarly to TCGA data; gene-sets were scored with singscore, stratified into high and low TC and LE scores using an optimized cut-point, and had their optimized hazard ratios and p-values calculated. Survival associations observed in TCGA OSCC patients were also replicated in TCGA pan-cancer datasets.<br><br>We replicate bioinformatics findings of two distinct CSC states using immunofluorescence staining of 3 tissue sections, confirming localization of the CD24 marker at the TC, and the CD44 marker at the LE.
Randomization	Given the exploratory nature of our research, allocating patients into experimental groups was not feasible/required.
Blinding	Due to the absence of experimental groups, no blinding was performed.

Reporting for specific materials, systems and methods

We require information from authors about some types of materials, experimental systems and methods used in many studies. Here, indicate whether each material, system or method listed is relevant to your study. If you are not sure if a list item applies to your research, read the appropriate section before selecting a response.

Materials & experimental systems

n/a	Involved in the study	
☐	☒	Antibodies
☒	☐	Eukaryotic cell lines
☒	☐	Palaeontology and archaeology
☒	☐	Animals and other organisms
☒	☐	Clinical data
☒	☐	Dual use research of concern

Methods

n/a	Involved in the study	
☒	☐	ChIP-seq
☒	☐	Flow cytometry
☒	☐	MRI-based neuroimaging

Antibodies

Antibodies used	Rabbit polyclonal anti-CD24 antibody (Abcam: 1:250 dilution; Catalog # ab244478) and mouse monoclonal anti-CD44 antibody (IM7, Invitrogen: 1:250 dilution; Catalog # 14-0441-82) as the primary antibodies. We included corresponding fluorophore-conjugated secondary antibodies goat anti-rabbit antibody conjugated to Alexa 546 and donkey anti-rat antibody conjugated to Alexa 488 (1:500; Jackson ImmunoResearch Laboratories) alongside Hoechst 33342 (1:1000).
Validation	Knockout validation was provided by the manufacturer, and dilution specifications were chosen according to manufacturer protocols.
